# Normative Misperceptions About Cannabis Use in a Sample of Risky Cannabis Users

**DOI:** 10.1177/11782218231166809

**Published:** 2023-04-06

**Authors:** John A Cunningham, Christina Schell, Nicolas Bertholet, Jeffrey D Wardell, Lena C Quilty, Alexandra Godinho

**Affiliations:** 1 National Addiction Centre, Institute of Psychiatry, Psychology and Neuroscience, Kings College London, London, UK; 2 Centre for Addiction and Mental Health, Toronto, ON, Canada; 3 Department of Psychiatry, University of Toronto, Toronto, ON, Canada; 4 Dalla Lana School of Public Health, University of Toronto, Toronto, ON, Canada; 5 Addiction Medicine, Department of Psychiatry, Lausanne University Hospital and University of Lausanne, Lausanne, Vaud, Switzerland; 6 Department of Psychology, York University, Toronto, ON, Canada

**Keywords:** Cannabis, normative misperceptions, Internet Intervention, Canada

## Abstract

**Introduction:**

This study examines normative misperceptions in a sample of participants recruited for a brief intervention trial targeting risky cannabis use.

**Methods:**

Participants who were concerned about their own risky cannabis use were recruited to help develop and evaluate intervention materials. At baseline, participants reported on their own cannabis use and provided estimates of how often others their gender and age used cannabis in the past 3 months. Comparisons were made between participants estimates of others cannabis use with reports of cannabis use obtained from a general population survey conducted during a similar time period.

**Results:**

Participants (N = 744, mean age = 35.8, 56.2% identified as female) largely reported daily or almost daily cannabis use (82.4%). Roughly half (55.3%) of participants estimated that others their age and gender used cannabis weekly or more often in the past 3 months, whereas the majority of people in the general population reported not using cannabis at all.

**Conclusions:**

Normative misperceptions about cannabis use were common in this sample of people with risky cannabis use. Limitations and possible future directions of this research are discussed, as well as the potential for targeting these misperceptions in interventions designed to motivate reductions in cannabis use.

**ClinicalTrials.org number:**

NCT04060602

## Introduction

People engaging in risky addictive behaviours often overestimate how often others engage in the same behaviour. Normative misperceptions have been observed in samples of people with unhealthy alcohol consumption, cigarette smoking and gambling.^1,2^ The current study sought to examine normative misperceptions among cannabis users. The study of normative misperceptions is important because the correction of these misperceptions^1-3^ using personalized normative feedback (PNF) interventions has been found to result in reliable, small-to-medium effects on changing behaviours,^4,5^ depending on the substance under investigation. Furthermore, the successful spread of PNF interventions from face-to-face interactions to other modalities, including telephone and internet, has the additional benefits of being scalable, cost-effective, anonymous and available on demand.^6,7^

There is some evidence of normative misperceptions in people who consume cannabis, although this research has primarily been conducted with college and university student samples.^1,8-14^ It is important to verify these findings in non-student groups, as these samples do not tend to accurately represent the general population and thus findings may not be generalizable.^15^ Furthermore, while PNF can be effective in reducing individual substance use and effect other health-behaviour change, limited research has investigated its effect on cannabis use.^4^ As such, an important component in the development and evaluation of a new PNF intervention is to establish whether normative misperceptions are occurring in the population under study. Thus, the current study sought to determine whether normative misperceptions occurred in a sample of participants who used cannabis in a risky fashion (as defined by a validated World Health Organisation scale; please see details in Methods section) and who had signed up to participate in a study developing and evaluating a personalized feedback intervention for cannabis. The sample was recruited in Canada after cannabis was legalized for use in adults 18 years and over.

## Methods

Online advertisements (Facebook, Kijiji) were used to recruit people who were: (1) concerned about their own cannabis use; and (2) interested in participating in a project to find ways to help people who were worried about their cannabis use. Details of the trial and the primary outcome results are published elsewhere.^16^ Briefly, participants were eligible for the trial if they were 18 years or older, lived in Canada, and reported risky cannabis use as measured by a score of 4 or more on the cannabis subscale of the Alcohol, Smoking and Substance Involvement Screening Test (ASSIST).^17^ As part of the baseline survey, participants provided information on their demographic characteristics and cannabis use (cannabis subscale of the ASSIST, frequency of use). Participants were subsequently asked 2 questions to assess their perceptions of other people’s cannabis use: (1) ‘How frequently do you think the average Canadian (*insert gender of participant*) your age used cannabis, marijuana or hashish in the past 3 months?’ (response options: never, less than monthly, monthly, weekly, daily or almost daily), and (2) ‘What percent of Canadians (*insert gender of participant*) your age do you think have not used cannabis, marijuana or hashish at all in the past 3 months?’ These outcomes were chosen to allow comparisons between participants’ estimates of others’ use and actual prevalence of use in the Canadian general population during the same time period the study was conducted using data from the 2019 Canadian Alcohol and Drugs Use Survey (CADS).^18^ The research was approved by the standing research ethics board of the Centre for Addiction and Mental Health.

## Results

Participants (N = 744) were recruited from September 2019 to March 2020. The large majority of participants (82.4%) reported using cannabis daily or almost daily in the past 3 months, while 12.8% reported weekly use, 4.2% reported monthly use and 0.7% reported less than monthly use. In addition, participants’ mean (SD) ASSIST score was 23.3 (10.3), indicating moderate risk associated with their cannabis use. The average (SD) age was 35.8 years old (12.8; age range: 18-76), 56.2% identified as female, 60.2% had some post-secondary education, 54.8% were full- or part-time employed and half (53.1%) reported a family income of CAD $20 000 or less.

**Figure 1.**
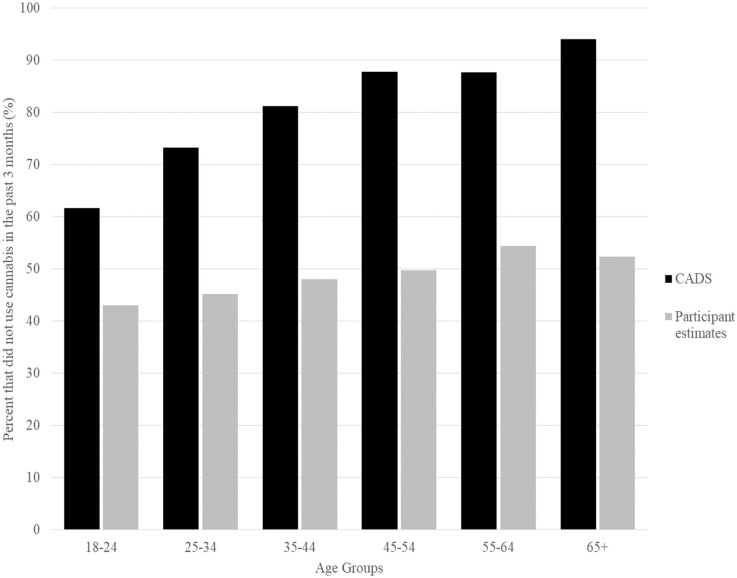
Percentage of Canadians by age group who did not use cannabis in the past 3 months according to the 2019 Canadian Alcohol and Drugs Use Survey (CADS) compared to participant estimates.

Participants estimates of the frequency other Canadians’ their age and gender used cannabis were compared to national averages from the CADS. While less than 10% of the general population reported using cannabis weekly or more often (ranged from 16.9% for males in the 18 to 24 age group to 1.8% in females 65 or older), more than half (55.4%) of participants in the current study believed that the average person their age and gender used cannabis weekly or more often. Participants were also asked what percent of people their age and gender did not use cannabis at all during the past 3 months. On average (SD) participants estimated that 47.1% (20.9%) of Canadians their age and gender did not use cannabis in the past 3 months. In comparison, the large majority of people in the Canadian population reported no cannabis use in the past 3 months (proportion ranged from 61% for males in the 18 to 24 age group to 95% in females 65 or older).^18^ Comparisons between participant estimates and national averages from the CADS by age group are summarized in Figure 1.

## Discussion

Normative misperceptions regarding cannabis use have been noted in previous research, although primarily in college student samples.^1,8-14^ The current study recruited a cannabis using, general population sample for an online intervention trial. Almost all participants in this study considerably overestimated the degree to which others their age and gender used cannabis.

It is important to identify the existence of normative misperceptions as they can be targeted in personalized feedback interventions with their correction theorized to motivate reductions in the target behaviour (in this case, cannabis use).^3^ In the present study, the personalized feedback intervention successfully modified participants’ normative perceptions.^16^ However, this correction did not result in concomitant reductions in cannabis use. More research is needed to identify whether it was some unique aspect of the current study (eg, method of recruiting, intervention content) that led to these negative findings. Alternatively, it may be that the correction of normative misperceptions may not act as a strong motivator for change among cannabis users as has been observed in other addictive behaviours. Lastly, it is possible that asking participants about their use and frequency of cannabis prior to asking about others’ use, may have led to an overestimation. Indeed, this has been observed among studies examining personal alcohol use versus perceptions of peer use in college students,^19^ and it is possible the same was observed for cannabis use in this study.

One limitation of this study was that the sample was not representative of the general population. The participants were recruited using an advertisement asking for people who were concerned about their cannabis use and were willing to help us evaluate an online intervention for cannabis. Further, the inclusion criterion required the presence of at least moderate risk cannabis use (as measured by the ASSIST). The resultant sample was frequent cannabis users, with the large majority reporting using daily or almost daily. It is possible that a representative sample would display less (or perhaps more) normative misperceptions about others’ cannabis use. Replication within a representative sample would also allow for the examination of normative misperceptions in groups with different socio-economic status (SES; it appears that the current sample may have a lower SES than would be observed in a representative sample). Further relevant topics for study include asking participants their perceptions of cannabis use in the general population as a whole (as opposed to those of the same age and gender), assessing perceptions (and the impact of norms) about people that the participant perceives as different from themselves and whether framing the questions within similar items about different drugs might have an impact on the accuracy of the norms provided. Also notable, the study was conducted in Canada, where cannabis was made legal about a year prior to the commencement of participant recruitment. This legalization may have led participants to assume that a larger proportion of people would be using cannabis. Further, it is unknown what the impact of recent changes in the legal status of cannabis would be on the efficacy of normative feedback interventions targeting the substance. More research is merited to estimate perceptions about others’ cannabis use in the general population and further, to determine the impact of legalization on interventions targeting social comparisons regarding its use.
